# Maternal Obesity-Associated Neonatal Morbidities in Early Newborn Period

**DOI:** 10.3389/fped.2022.867171

**Published:** 2022-05-25

**Authors:** Azima Kureshi, Rubia Khalak, Jamie Gifford, Upender Munshi

**Affiliations:** Department of Pediatrics, Albany Medical College, Albany, NY, United States

**Keywords:** transient tachypnea of newborn, respiratory distress syndrome, neonatal hypoglycemia, maternal obesity, cesarean

## Abstract

Maternal obesity has been associated with pregnancy-related complications and neonatal morbidities. The primary aim of this study was to evaluate early neonatal morbidities associated with maternal obesity from the infant-mother dyad data set at a single, large Regional Perinatal Center (RPC) in NY. A retrospective chart review of all mother-infant dyads born from January 2009 to December 2019 was done. Maternal obesity was defined using the NIH definition of pre-pregnancy body mass index (BMI) ≥ 30 Kg/m^2^. Maternal data included pre-pregnancy BMI, gestational diabetes, hypertension, and mode of delivery. Neonatal data recorded the birth weight, gestational age, respiratory support after delivery, diagnosis of transient tachypnea of the newborn (TTN), respiratory distress syndrome (RDS), neonatal hypoglycemia (NH), and hypoxic-ischemic encephalopathy (HIE). Diagnosis of TTN, RDS, NH, and HIE was defined by the service neonatologist and cross-checked by the data system review neonatologist. Medical records of 22,198 infant-mother dyads included in the study had 7,200 infants (32.4%) born to obese mothers and 14,998 infants (67.6%) born to non-obese women. There was a statistically significant increase in the diagnosis of gestational diabetes, gestational hypertension, and cesarean deliveries in obese mothers. Diagnosis of TTN, RDS, and NH was significantly higher in infants born to obese mothers, while HIE incidence was similar in both the groups. Infants born to obese mothers are more likely to be delivered by cesarean section and are at a higher risk of diagnosis of transient tachypnea of newborn, respiratory distress syndrome, and hypoglycemia in the early neonatal period.

## Introduction

The incidence of obesity has increased worldwide over the past few decades, especially in developed countries, and it has been linked to major public health problems of diabetes and hypertension (1, 2). Obesity has affected women of childbearing age proportionally and is currently the most prevalent problem in pregnancy that may affect both the mother and her newborn (3). Pregnant women may experience short- and long-term consequences because of their obesity; specifically, they are more often diagnosed as having gestational diabetes, pregnancy-induced hypertension, preeclampsia, preterm labor, venous thromboembolism and at delivery, tend to require instrumental assistance or cesarean section more often than their non-obese counterparts (4–6). Most of these maternal morbidities related to obesity can negatively impact the health of the fetus by producing an obesogenic intrauterine environment that delays lung maturity (7) and places the newborn at a higher risk of transient tachypnea of the newborn (TTN) or surfactant deficiency, leading to respiratory distress syndrome (RDS) (3, 8, 9). Glucose intolerance or gestational diabetes associated frequently with maternal obesity increases the risk of fetal hyperinsulinemia and abnormal glucose homeostasis in the newborn. Fetal hyperinsulinemia also predisposes to fetal macrosomia, which may cause difficult labor requiring instrumentation or surgery, and increases the chances of birth injuries and risk of hypoxic-ischemic encephalopathy (HIE) (10, 11). There have been several studies reported in the obstetric literature that highlight the complications of obesity in pregnant women (12); however, the impact of maternal obesity on early neonatal outcomes has not been extensively studied in a large cohort (13). Obesity in pregnancy poses challenges for both the mother and her offspring; there are increased awareness and anticipation of maternal complications with recommendations from obstetric professional bodies (14, 15), but similar awareness and anticipation of newborn problems posed by maternal obesity may not be fully realized by newborn caretakers. We undertook this investigation with the primary aim to study the risk of respiratory transition, glucose homeostasis, and hypoxic-ischemic encephalopathy in newborn babies delivered by obese mothers at our center for more than a decade.

## Methods

Deidentified maternal and neonatal data were acquired from the New York State Perinatal Database related to all the live births at Albany Medical Center from January 2009 to December 2019, born at a gestation of 23 to 41 completed weeks. A retrospective chart review of all infants and their mothers was conducted, and the study was approved by the Albany Medical Center Institutional Review Board. Maternal data included pre-pregnancy BMI, pre-pregnancy diabetes, gestational diabetes, pre-pregnancy hypertension, gestational hypertension, and mode of delivery. Neonatal data recorded the birth weight, gestational age at birth, prematurity (gestational age < 37 weeks at birth), admission to the neonatal intensive care unit (NICU), and the need for respiratory support in the form of high flow nasal cannula (HFNC), continuous positive airway pressure (CPAP), or non-invasive intermittent positive pressure ventilation (nIPPV), all termed as non-invasive respiratory support and placement of endotracheal tube with mechanical ventilation termed as invasive respiratory support. Neonatal morbidities, such as transient tachypnea of the newborn (TTN), were defined as the need for non-invasive respiratory support after delivery and improving <72 h of age. Diagnosis of respiratory distress syndrome (RDS) was defined by non-invasive respiratory support for > 72 h along with chest x-ray findings consistent with RDS, an increased fraction of inspired oxygen (FiO_2_) > 0.21, and/or invasive mechanical ventilation *via* an endotracheal tube with or without surfactant use. Blood glucose screening was done by a heel stick within the first few hours of life as per protocol in the newborn nursery for infants of diabetic mothers, small or large for gestational-age infants and stable “late preterm infants” between 35 and 0/7 weeks to <37 weeks gestation, and all newborn infants admitted to NICU upon admission. Neonatal hypoglycemia (NH) was defined as blood glucose <40 mg/dl treated with either oral dextrose gel or intravenous dextrose. Hypoxic-ischemic encephalopathy (HIE) was defined as fetal acidosis on cord pH <7.1 or within the first hour, base excess worse than 12 meq/L, the need for active delivery room resuscitation and abnormal neurological examination, and meeting our neonatal intensive care unit criteria for starting therapeutic hypothermia protocol. The exclusion criteria included major chromosomal abnormalities, congenital anomalies, and stillbirths. Mothers were categorized into obese and non-obese groups with maternal obesity defined as BMI ≥ 30 kg/m^2^ per National Institute of Health (NIH) criteria. Infants born to obese and non-obese groups were then compared using Stata version 12.1 by Statacorp. Students *t*-test was used to compare normally distributed continuous data but chi-square test compared nominal data. Binary logistic regression evaluated the effect of maternal obesity on respiratory distress while controlling for gestational age, birth weight, maternal diabetes, and hypertension.

## Results

**Figure 1:**
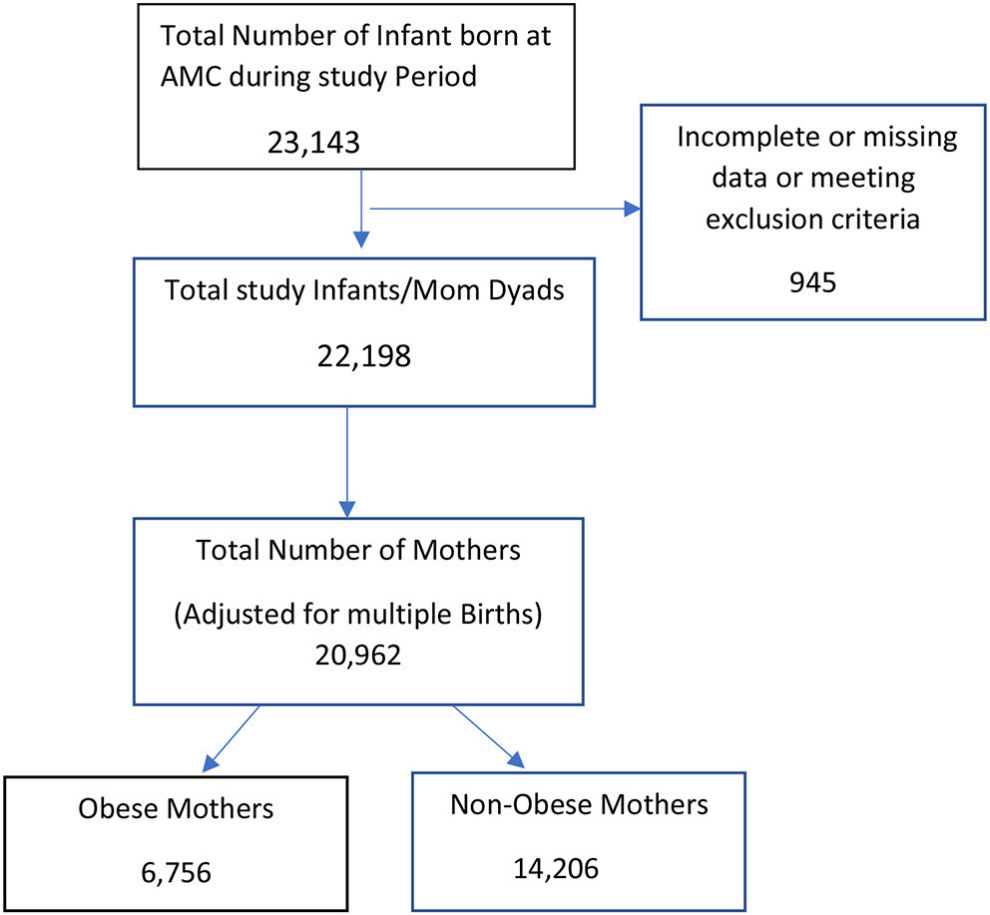
Case flow diagram.

Medical records of 23,143 infants and mothers were reviewed; 945 dyads were excluded due to incomplete or missing data or meeting the exclusion criteria. A total of 22,198 mother-infant dyads were included in the final analysis. Of the 22,198 infants included in this study 7,200 (32.4%) infants were born to 6,756 obese mothers, while 14,998 (67.6%) infants were born to 14,206 non-obese mothers, accounting for multiple births (see Figure 1). Maternal morbidities prior to and during the pregnancy were reviewed. Obese mothers were more likely to experience pre-pregnancy diabetes (5.4 vs. 1.9%) and subsequent gestational diabetes (18.9 vs. 7.6%, *p* < 0.001) than non-obese mothers. Obese mothers were also more likely to experience pre-pregnancy hypertension (12.8 vs. 2.8%) and gestational hypertension (16.1 vs. 13.7%, *p* <0.001) than mothers who were not obese. Cesarean deliveries were significantly higher in obese mothers (52.9%) than in non-obese mothers (37.6%, *p* < 0.001; Table 1). The infants were categorized by maternal BMI and analyzed for their demographics and neonatal morbidities. The infants of obese mothers were delivered at a lower gestational age than the infants of non-obese mothers (37.1 ± 3.6 vs. 37.5 ± 3.4 weeks, *p* <0.001), and their birth weight (3,077 ± 879 g) was higher than the infants of non-obese mothers (3,035 ± 797 g, *p* < 0.001). The prematurity birth rate defined as birth <37 weeks was significantly increased in the infants of obese mothers (27.4% vs. 23.5%, *p* < 0.001). The infants of obese mothers had a statistically significant higher incidence of TTN (14.3% vs. 11.6%, *p* < 0.001) than the infants of non-obese mothers, and they developed respiratory distress syndrome (7.2% vs. 6.1%, *p* = 0.002) at a higher rate than infants of non-obese mothers. The infants born to obese mothers were more likely to develop neonatal hypoglycemia than the infants born to non-obese mothers (4.4% vs. 2.5%, *p* < 0.001). Logistical regression analysis revealed a statistically significant increase in respiratory distress syndrome (*p* = 0.01) and neonatal hypoglycemia (*p* = 0.05) in the infants of obese mothers when adjusted for pre-pregnancy and gestational maternal diabetes and hypertension. The incidence of hypoxic-ischemic encephalopathy was similar in both groups (0.2% vs. 0.2%, *p* = 0.8) (see Table 2).

**Table 1:** Maternal Morbidity by Body Mass Index (BMI) as kg/m^2^.

**Maternal Morbidity *N* = 20,962 (Adjusted for multiple births)**	**Obese Mothers (BMI ≥30) *n* = 6,756**	**Non-Obese Mothers (BMI <30) *n* = 14,206**	***P*** -value*
Pre-pregnancy diabetes (%)	371 (5.5)	273 (1.9)	<0.001
Gestational diabetes (%)	1,261 (18.7)	1,055 (7.4)	<0.001
Pre-pregnancy hypertension (%)	856 (12.7)	398 (2.8)	<0.001
Gestational hypertension (%)	1,421 (21.0)	1,858 (13.1)	<0.001
Cesarean delivery (%)	3,474 (51.4)	5,083 (35.8)	<0.001

* *Groups compared by Chi Square test*.

**Table 2:** Infant demographics and Early Neonatal Morbidity by Maternal BMI (kg/m^2^).

**Demographic and clinical characteristics of infants** ***N*** **= 22,198**	**Infants of Obese Mothers (BMI ≥30)** ***n*** **= 7,200**	**Infants of Non-Obese Mothers (BMI <30)** ***n*** **= 14,998**	***P*** **-value**
GA (weeks) Mean ± SD	37.1 ± 3.6	37.5 ± 3.4	<0.001*
Birth weight (g) Mean ± SD	3,077 ± 879	3,035 ± 797	<0.001*
Prematurity, GA <37 wks (%)	1,970 (27.4)	3,527 (23.5)	<0.001
Admission to NICU (%)	1,796 (24.9)	3,171 (21.1)	<0.001
Non-Invasive Resp. Support (%)	1,094 (15.2)	1,895 (12.6)	<0.001
Mechanical ventilation (%)	366 (5.1)	717 (4.8)	0.32
TTN (%)	1,029 (14.3)	1,736 (11.6)	<0.001
RDS (%)	516 (7.2)	909 (6.1)	0.002
Neonatal Hypoglycemia (%)	320 (4.4)	379 (2.5)	<0.001
HIE (%)	14 (0.2)	31 (0.2)	0.8

*Denotes t test, all other comparisons were done by Chi^2^ test*.

## Discussion

Our study confirms the finding that obese mothers have a disproportionately higher incidence of diabetes and hypertension prior to and during pregnancy than non-obese mothers. They were also at a higher risk for requiring cesarean deliveries than the non-obese mothers. Neonates born to obese mothers were delivered at an earlier gestational age and at higher birth weight than their counterparts. They also received respiratory support after the delivery room due to delayed respiratory transition, in addition to developing respiratory distress syndrome as seen in prematurity and neonatal hypoglycemia. A recent study by Lindberger et al. has shown mothers with central adiposity in early to mid-pregnancy within a year prior to conception experienced more cesarean deliveries and delivered babies at an increased birthweight. This supports our findings that obese mothers had a higher rate of cesarean deliveries and delivered larger babies than non-obese mothers and increased risk of NICU admission, substantiating our study that infants born to obese mothers delivered neonates who were born at an earlier gestational age and suffered more complications that needed admission to NICU (16). In another study by Mortier et al., gestational diabetes was found to be an independent risk factor in neonatal severe respiratory distress syndrome in neonates born after 34 weeks (9). Our observation of increased association of gestational diabetes in obese mothers and respiratory distress syndrome in their offsprings agrees with their report. McGillick et al. (8) explain the multifactorial mechanisms of “obesogenic” intrauterine environment that leads to delay in fetal lung maturation in a detailed review. This can cause difficulty in the extrauterine respiratory transition and predispose to early respiratory distress, needing respiratory support and admission to neonatal intensive care unit. Obesogenic intrauterine environment can also explain the risk of increased fetal insulin production that may increase the risk of neonatal hypoglycemia. Our finding of increase in neonatal hypoglycemia has been reported by Turner et al. (17) along with increase in the incidence of cesarean section in their obese mothers. On a logistic regression, while controlling for birth weight, gestational age, maternal diabetes, and hypertension, being born to obese mother was an independent risk factor in development of respiratory distress syndrome and neonatal hypoglycemia. Fetal macrosomia and difficult delivery in maternal obesity increase the risk of birth injury and hypoxic ischemic encephalopathy in newborn as reported by Khalak and Horgan (10) and Perssonet et al. (11): both reports were population based with a mix of community hospitals and regional centers. Despite the increased risk obesity poses on a mother and her newborn, we did not observe increase in HIE diagnosis in infants born to obese mothers at our center. It may be noteworthy that most infants with a diagnosis of HIE admitted to our RPC for therapeutic hypothermia were out born, raising a concern of optimal obstetric interventions and newborn resuscitative measures that may not be always available in a community care hospital setting (10).

There were few limitations to this study. The design of this study was retrospective in nature, and the neonatal diagnoses of TTN, RDS, NH, and HIE were determined by the service neonatologist based on our practice parameters in our unit over the study period. The strengths of our study included a large sample size and a longitudinal study period of 11 years at a single health care center. In addition, our study was conducted at a large and diverse population-based perinatal center; therefore, the findings should be applicable to the general population in the country. We conclude that there is an increase in morbidities in infants born to obese mothers in the early neonatal period, including delayed respiratory transition, respiratory distress syndrome due to respiratory immaturity, and neonatal hypoglycemia. Our study highlights the early neonatal morbidities in infants born to obese mothers that should be anticipated by the newborn care providers and discussed during prenatal consultations with the mothers/family and their care providers. Awareness and anticipation of these early neonatal morbidities can also help newborn care providers to prepare, utilize appropriate resources, and provide optimal care for the newborn.

## Data Availability Statement

The raw data supporting the conclusions of this article will be made available by the authors, without undue reservation.

## Ethics Statement

The studies involving human participants were reviewed and approved by Albany Medical Center IRB. Written informed consent from the participants' legal guardian/next of kin was not required to participate in this study in accordance with the national legislation and the institutional requirements.

## Author Contributions

UM conceived the research project and methods, analyzed the results, revised the first draft, and edited the manuscript. AK collected the data, analyzed the data, and wrote the first draft of manuscript. JG collected and analyzed the data. RK suggested the methods, analyzed the data, and edited the manuscript. All authors contributed to the article and approved the submitted version.

## Conflict of Interest

The authors declare that the research was conducted in the absence of any commercial or financial relationships that could be construed as a potential conflict of interest.

## Publisher's Note

All claims expressed in this article are solely those of the authors and do not necessarily represent those of their affiliated organizations, or those of the publisher, the editors and the reviewers. Any product that may be evaluated in this article, or claim that may be made by its manufacturer, is not guaranteed or endorsed by the publisher.
